# Removal of Maxillary Sinus Metallic Foreign Body Like a Hand Sewing Needle by Magnetic Iron

**DOI:** 10.5005/jp-journals-10005-1237

**Published:** 2014-04-26

**Authors:** Linqin Shao, Xiurong Qin, Yingwei Ma

**Affiliations:** Chairman, Department of Pediatric Dentistry, Jinan Stomatologic Hospital, Shandong Province, China; Assistant Professor, Department of Pediatric Dentistry, Jinan Stomatologic Hospital, Shandong Province, China; Associate Professor, Department of Stomatology, Jinan Central Hospital Affliated to Shandong University, Shandong, China

**Keywords:** Maxillary sinus, Foreign body, Spiral cone-beam computed tomography

## Abstract

Metallic foreign bodies are rarely found in the maxillary sinus, and usually they have a dental origin. Two main surgical app­roaches are currently used for the removal of foreign bodies in the maxillary sinus: the bone flap and the endoscopic sinus tech­niques. However, the treatment is not only surgical removal. We are reporting one case of foreign body like a hand sewing needle entered into the maxillary sinus through an unusual route— carious deciduous molar tooth. It was diagnosed by three-dimensional images from cone-beam computed tomography (CBCT) and removed by a simple procedure, with magnetic iron, thereby avoiding the risk of damage to a large portion of the alveolar bone near the maxillary sinus.

**How to cite this article: **Shao L, Qin X, Ma Y. Removal of Maxillary Sinus Metallic Foreign Body Like a Hand Sewing Needle by Magnetic Iron. Int J Clin Pediatr Dent 2014;7(1):61-64.

## INTRODUCTION

Metallic foreign bodies are rarely found in the maxillary sinus, and usually they have a dental origin.^1^ There is a possibility of foreign bodies penetrated into the maxillary sinus due to the special features of the posterior aspect of the maxillary bone. In this area, the bone is poor quality and quantity.^2^ Secondary, local lesion, such as maxillary sinusitis, tooth with apical lesion, dentigerous cyst and tumor, may cause resorption of surrounding bone. This anatomical condition along with the low density of the maxillary bone, severe atrophy, bone resorption, changes in the intranasal pressure, occlusal forces and lack of experience from the dental implant surgeon may be responsible for the foreign bodies in the maxillary sinus.^3,4^ Foreign bodies displaced into the maxillary sinus must be removed because they can disturb the mucocilliary function and cause potential compli­cations, mainly acute or chronic maxillary sinusitis.^5,6^ For this reason, foreign bodies should be diagnosed and removed on time as well as it occurs with other metallic foreign bodies, in order to prevent complications.

Various surgical techniques for the removal of a foreign body from maxillary sinuses have been reported. Treatment modalities to address this complication include removal of the implant either through endonasal endoscopic surgery or with an intraoral approach, through the anterior maxillary wall. In some cases these two methods can be combined.^7^ The choice of procedure depends on the time elapsed from the foreign body into the maxillary sinus, the existence of oroantral communication, and the patient's choice, as endoscopic procedures are usually performed under general anesthesia, while local anesthesia can be applied for intraoral removal, as an in office procedure.^2^ However, all of the both treatment options are surgical challenges due to a combination of difficulty in access and close anatomical relationship to vital structures.

However, the treatment is not only surgical removal. We are reporting one case of foreign body like a hand sewing needle entered the maxillary sinus through an unusual route—carious deciduous molar tooth. It was diagnosed by three-dimensional images from cone-beam computed tomography (CBCT) and removed by a simple and quick procedure, with magnetic iron, thereby avoiding the risk of damage to a large portion of the alveolar bone near the maxillary sinus.

**Fig. 1:**
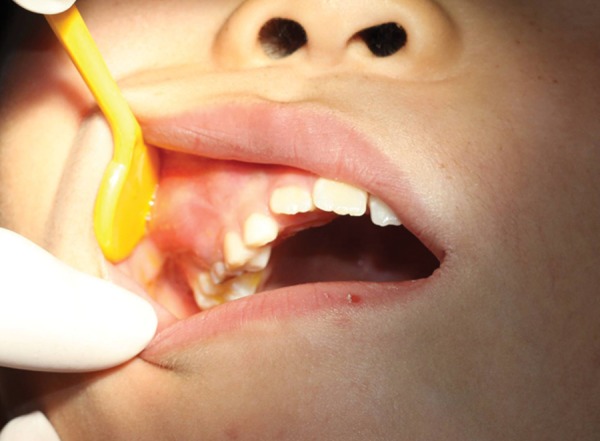
Primary maxillary right first molar (Tooth A) was seriously carious and serious red swelling of the buccal mucosa

**Fig. 2:**
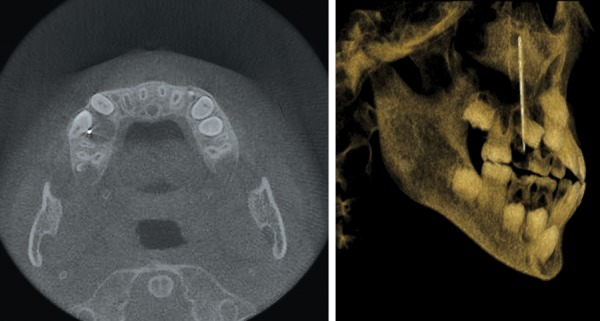
CBCT scan revealed a 4.2 cm long hyperdense image with well-defined margins within the right maxillary sinus, associated with a dentigerous cyst with the crowns of permanent maxillary right second premolar (Tooth 4) germ, the tooth germ displace

## CASE REPORT

**Fig. 3:**
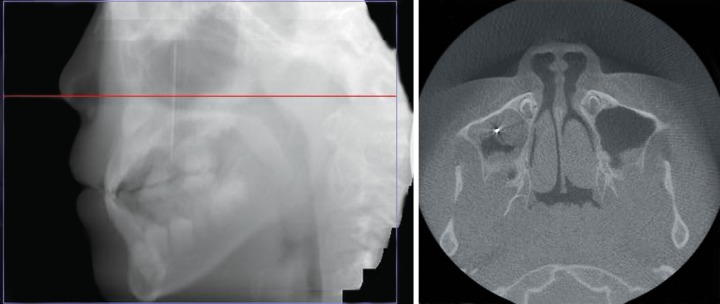
CBCT scan revealed a hyperdense image within the right maxillary sinus, and the sinusal mucosa was thicker than the normal left side

A 10-year-old male patient, consulted for one needle entered into his tooth for 1 day. The patient had many carious teeth. He felt uncomfortable after eating because of the food impaction. He often removed the impacted food debris with a hand sewing needle. One day before, when he used the hand sewing needle, he found the needle entered into the carious tooth and disappeared. At consultation, primary maxillary right first molar (Tooth A) was seriously carious and serious red swelling of the buccal mucosa (Fig. 1). The hand sewing needle was not seen in the tooth. And, the patient presented with half a year history of headache and right-sided maxillary discomfort that was associated with nasal congestion. A three-dimensional images from CBCT scan revealed a 4.2 cm long hyperdense image with well-defined margins within the right maxillary sinus (Fig. 2). The sinusal mucosa was thicker than the normal left side (Fig. 3). CBCT scanning also showed a low density shadow around the apical regions and under the furcation area of Tooth A. And, a dentigerous cyst with the crowns of permanent maxillary right second premolar (Tooth 4) germ, the tooth germ displaced into ectopic position (Fig. 2). The diagnosis was a maxillary sinus foreign body, maxillary sinusitis, periapical periodontitis of Tooth A and dentigerous cyst of Tooth 4.

**Figs 4A and B:**
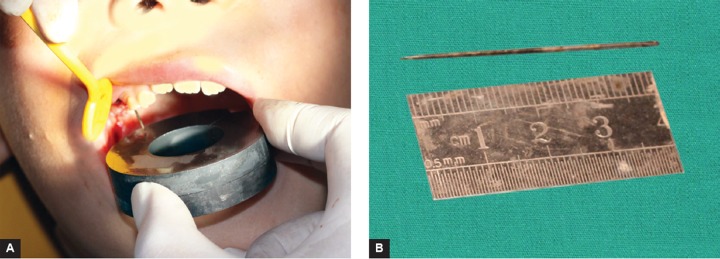
The sewing needle was attracted out by magnetic iron

**Fig. 5:**
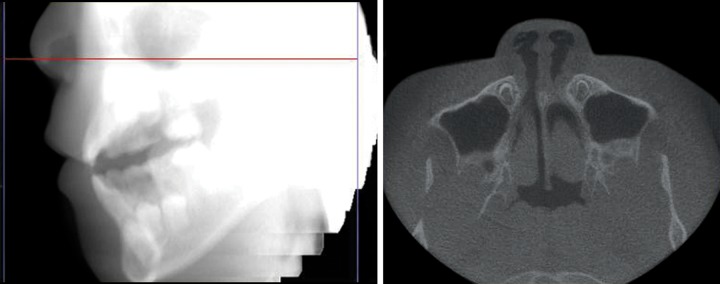
CBCT scan image revealed normal mucosal thickness and no opacification of the right maxillary sinus 2 months after the removal of needle

### Surgical Procedure

The patient was referred for the extraction of Tooth A and removal of the foreign body. In this case report, the foreign body was removed by a simple procedure, with magnetic iron. Tooth A was extracted under local anesthesia by Pri-macaine. The dentigerous cyst curettage was performed and a lot of normal saline was irrigated. The probing depth was 4 cm into the alveolar fossa, using the modified probe, but the dentist can not touch the sewing needle. To prevente a large portion of the alveolar bone near the maxillary sinus, an idea was accidentally thought to remove the foreign body with magnetic iron. So the dentist found a magnetic iron and had a try. The method was good and the sewing needle was attracted out (Figs 4A and B). Lots of normal saline was irri­gated for the alveolar fossa to prevente wound infection, and then packed with iodoform gauze. Dressing change once per week for a month. Antibiotics were prescribed and the recovery was uneventful.

### Follow-up

The postoperative course was uneventful for the patient without headache and any clinical signs of acute or chronic sinusitis. A CBCT scan image revealed normal mucosal thickness and no opacification of the right maxillary sinus, 2 months after the removal of needle (Fig. 5).

## DISCUSSION

Among all maxillary sinusitis surgically treated, around 5 to 15% are caused by foreign body of dental origin.^8^ The typical bodies described are: dental implants and dental roots. However, the hand sewing needle has been rarely reported. In this case report, many reasons for the needle entered into the maxillary sinus. These local infections, such as Tooth A with periapical periodontitis, dentigerous cyst of Tooth 4 and the maxillary sinusitis caused resorption of surrounding bone. When the patient used a hand sewing needle to remove the impacted food debris, the sharp needle tip along with the little boy's inappropriate force easily penetrated the low quality bone and mucous membrane into the maxillary sinus.

Foreign bodies into the maxillary sinus may cause infectious complications due to the contact of the foreign body with the mucosa of the sinus interior, mainly, acute or chronic sinusitis.^5,6^ Local infection around the area may cause resorption of surrounding bone in the future. In this case report, although the boy had no acute symptoms of maxillary sinusitis, the foreign body should be diagnosed and removed on time to prevent any other complications. CBCT scan was used to confirm the diagnosis, determining the size and the exact location of the hand sewing needle.

Various surgical techniques for the removal of a foreign body from maxillary sinuses have been reported. Two main surgical approaches are currently used for the removal of foreign bodies in the maxillary sinus: the bone flap and the endoscopic sinus techniques.^1^ The best treatment option to remove displaced implants into the maxillary sinus is the functional endoscopic sinus surgery. The advantage of this surgery—apart from removal of the displaced implant from the maxillary sinus—is the creation of adequate patency from the natural maxillary ostium.^9,10^ Even though func­tional endoscopic sinus surgery presents extremely limited complications, it requires the application of general anes­thesia, and in certain cases where oroantral communication exists, it must be combined with the intraoral approach.^2^However, the treatment is not only surgical removal, in this case report, the foreign body was removed by a simple procedure, with magnetic iron. There were two factors for choosing this technique, one was that the time elapsed from the foreign body into the maxillary sinus was short so the hand sewing needle was not wrapped with fiber, the other one was that the needle did not shift in the maxillary sinus, so it was attracted out along the original route by the magnetic iron.

## CONCLUSION

Metallic foreign body into maxillary sinus is an uncommon but possible event. Therefore, it is important to accurately evaluate the specific characteristics of the patient and the foreign body. This is the first case offering a removal of maxillary sinus metallic foreign body like a hand sewing needle by magnetic iron. This technique is simple, comfortable for the patient and it can be performed under local anesthesia without special equipment, including endoscopy. What is more, it could avoid the risk of damage to a large portion of the alveolar bone near the maxillary sinus.
